# Beliefs in conspiracy theories and the need for cognitive closure

**DOI:** 10.3389/fpsyg.2013.00378

**Published:** 2013-06-27

**Authors:** Patrick J. Leman, Marco Cinnirella

**Affiliations:** Department of Psychology, Royal Holloway, University of LondonLondon, UK

**Keywords:** bias, cognitive closure, conspiracy theory, evidence, knowledge

## Abstract

An important component of conspiracy theories is how they influence, and are influenced by, the evaluation of potential evidence. Some individuals may be more open minded regarding certain explanations for events whereas others may seek closure and thus cut off a conspiracy explanation. Two studies examined the relationship between the need for cognitive closure (NFCC), levels of belief in real world conspiracy theories, and the attribution of conspiracy theories to explain events. A first, small (*N* = 30) and preliminary study found no relationship between NFCC and beliefs in conspiracy theories, suggesting that both advocates and opponents of conspiracy explanations do not differ on this dimension. A second study (*N* = 86) revealed that evidence for and against conspiracy theories had an influence on attributions of the likelihood of a conspiracy to explain a novel event. Specifically, after reading evidence individuals with high levels of belief in conspiracy theories tended to rate a conspiracy explanation as more likely whereas those with low levels of belief rated it as less likely. However, when the need for cognitive closure (NFCC) was experimentally lowered the effects of prior beliefs in conspiracy theories diminished.

Conspiracy theorists often argue that official accounts of events “close off” the possibility of alternative explanations by misinterpreting or ignoring evidence (e.g., Posner, 1993; Pipes, 1997; Pastore, 2004). In contrast, those who argue against conspiracy theory accounts frequently suggest that such accounts do not bear up to rigorous scrutiny from a scientific or rational perspective (Clarke, 2002). In the present research we explore, for the first time, the relationship between beliefs in conspiracy theories and the need for cognitive closure (NFCC, Webster and Kruglanski, 1994). We also investigate a related question of how evidence affects the attribution of the likelihood that a conspiracy theory explains a novel event.

The factors that underpin beliefs in conspiracy theories—broadly defined as a set of beliefs that are used to explain how a group of individuals is covertly seeking to influence or cause certain events—constitute fertile ground for psychological study. Not only are beliefs in conspiracy theories widespread and on the increase (e.g., Goertzel, 1994; Swami et al., 2011), they are also prone to a third person effect whereby we feel others believe in conspiracy theories more than we do (Douglas and Sutton, 2008). Conspiracy beliefs also have profound importance in a society where conspiracy accounts are implicated in erroneous interpretations of important events (Leman and Cinnirella, 2007), may be associated with mistrust of political and social institutions (Kramer, 1999), and affect behavior such as the decision whether to pursue health care (Bird and Bogart, 2003; Tickner et al., 2010) or cooperate with the criminal justice system (Parsons et al., 1999). It is somewhat surprising then, that with some notable exceptions (e.g., Graumann and Moscovici, 1987; Swami et al., 2011), comparatively few studies have sought to examine factors and processes that are associated with beliefs in conspiracy theories.

Social psychologists often argue that beliefs in conspiracy theories are connected with broader social and intergroup conflicts where conspiracy theories are used to justify and maintain conflict or to attribute blame to an unjust social system (Crocker et al., 1999). Other research has sought to explain the appeal of conspiracy theories by focusing on personality characteristics of conspiracy theorists. Among other factors, a sense of *powerlessness* and *anomie*—an inability to affect change and feelings of insignificance within society—have been found to correlate positively with high levels of beliefs in conspiracy theories (Hamsher et al., 1968; Whitson and Galinsky, 2008; Bruder et al., 2013).

In terms of other aspects of personality, the picture appears more complex. McHoskey (1995) found a negative relationship between authoritarian attitudes and endorsements of conspiracy theories (arguing that individuals with authoritarian attitudes are more likely to perceive Government as legitimate and morally inscrutable). Individuals with a high score on the Right-Wing Authoritarianism Scale (Altemeyer, 1988) were more dismissive of possible conspiratorial explanations However, in contrast (Abalakina-Paap et al., 1999), found a positive relationship between right-wing authoritarianism and conspiracy beliefs. Political orientation and beliefs may influence conspiracy beliefs in different ways in different contexts. In this vein, Swami (2012) found a positive relationship between right wing authoritarianism and beliefs in anti-Jewish conspiracy theories, but a negative relationship with general beliefs in conspiracy theories. Individuals may well pick and choose theories that fit with a particular political view or belief system (e.g., Leman, 2007; Wood et al., 2012).

Beliefs in conspiracy theories also have much to do with the ways in which individuals interpret and contest the legitimacy of evidence (e.g., Harrison and Thomas, 1997; Leman, 2007). For instance, heuristics such as the linking of a major event with a major cause may account for the attribution of conspiracy theories to explain major public events (Leman and Cinnirella, 2007). Individuals may be reluctant to consider or assimilate disconfirming evidence once conspiratorial beliefs have become established (confirmation bias, e.g., Klayman and Ha, 1987). However, the same resistance to novel or contradictory evidence can be observed across different domains of reasoning, and the same psychological processes of resistance to contradictory information could just as likely apply to anti-theorists too. It is also evident that disconfirming evidence can be interpreted in different ways. While high quality empirical evidence will generally resolve disagreement (Lord et al., 1979), ambiguous or questionable evidence is prone to an interpretation based on confirmation heuristics (see again Klayman and Ha, 1987) and a desire to avoid dissonance (Festinger, 1957). Moreover, evidence that is deemed to confirm an individual's existing beliefs will tend to be unquestioned and accepted whereas disconfirming evidence will often be critically evaluated and rejected (see again Lord et al., 1979). This serves to reduce cognitive dissonance between attitude and evidence. As a result the same information can often be appropriated to support both sides of an argument.

Other variables such as NFCC may influence the motivational heuristics responsible for interpreting evidence. Previously, researchers have identified relationship between tolerance of ambiguity and beliefs in conspiracy theories (Abalakina-Paap et al., 1999). However, NFCC is a subtly different concept in that is identifies a drive for a certain view involving preference for order and structure, as well as discomfort with ambiguity, and closed mindedness (Webster and Kruglanski, 1994). It involves two basic tendencies. Firstly it involves a desire to obtain a quick solution or closure: this is referred to as seizing. Secondly it involves a tendency to preserve this solution, thus maintaining closure: referred to as friezing. Research has found that NFCC is both dispositional and situational and therefore open to manipulation. Time constraints (De Grada et al., 1999) and cognitive load (Ford and Kruglanski, 1995) can increase levels of need. Inversely, heightened accountability reduces the level of need (Ford and Kruglanski, 1995). This reduction relies on the fact that when making important decisions, the tendency to seize on a quick answer will be negated.

A high level of NFCC produces a reliance on confirmation heuristics (De Dreu et al., 1999) that results in a strengthening of existing beliefs. Low level NFCC induces systematic processing (Klein and Webster, 2002) resulting in greater scrutiny of information and evidence. Thus, levels of NFCC determine how information may be processed, understood, and accepted when interpreting evidence. For instance, Kruglanski et al. (1993) found that participants under high NFCC conditions are less persuadable than those low in NFCC. NFCC identifies a universal psychological process and in this respect should extend to how conspiracy theorists and non-theorists process evidence and develop beliefs.

The present research comprised two studies. Taken together these studies explored, for the first time, the ways in which NFCC relates to levels of belief in conspiracy theories and the attribution of a conspiracy theory to explain novel events. The first study sought to explore the correlation between various personality characteristics, including NFCC, and beliefs in conspiracy theories. Our key aim in the first study was to clarify the relationship between NFCC and belief in conspiracy theories. We predicted, given that NFCC denotes a general psychological process, that there is would be no association between NFCC and levels of belief in conspiracies. Our second study examined the relationship between NFCC, beliefs in conspiracy theories and interpretation of evidence. This second study built upon the first by focusing on how different factors may affect judgments about a novel, ambiguous event. Specifically, a core question is how NFCC, beliefs in conspiracy theories, and different types of evidence affect judgments of the likelihood that the event was the result of a conspiracy theory.

## Study 1

A key motivation for our first study was to explore the relationship between NFCC and levels of belief in conspiracy theories. This was a small scale preliminary study to establish whether correlations exist between several key variables and NFCC. Webster and Kruglanski (1994) argue that NFCC is both dispositional and situational. Individuals with a high NFCC tend to be more entrenched in their attitudes and seek to reach a decision or make a judgment more quickly and with less scrutiny than those with low NFCC. In this first study, NFCC we treated as dispositional and expected no relationship between NFCC and levels of belief in conspiracy theories. In other words, we hypothesized that both conspiracy theorists and non-theorists (anti-theorists) can employ rigid, dogmatic, and a “closed approach” in evaluating evidence.

We also examined relationships between authoritarianism, interpersonal trust and alienation, as well as the attribution of the likelihood of a conspiracy theory to explain events surrounding a fictitious scenario involving the death of a President in a plane crash. This last item was used to assess how far individuals were inclined to attribute a conspiracy theory to account for a novel situation.

Based on the previously reported work of (e.g., McHoskey, 1995; Swami, 2012), there is a somewhat complex relation between authoritarianism and beliefs in conspiracy theories. We therefore tentatively predicted that there would be a negative relationship between levels of authoritarianism and beliefs in conspiracy theories and the attribution score. Similarly, previous research has suggested that levels of interpersonal trust are negatively related to beliefs in conspiracy theories (Goertzel, 1994). Hence it was predicted that there would be a negative relationship between levels of interpersonal trust and beliefs in conspiracy theories. However, no correlation was predicted between levels of interpersonal trust and the attribution score.

Finally, an alienation scale (Ray, 1982) was also employed. This reflected an attempt to broaden research in the area. The scale measured factors relating to both powerlessness and anomie, but in line with general feelings of alienation. It was predicted that there would be a positive relationship between alienation scores and scores on the conspiracy and attribution scales, in line with findings reported above on previous work exploring anomie and powerlessness (Hamsher et al., 1968; Goertzel, 1994; Abalakina-Paap et al., 1999).

Based on previous research we predicted that gender would not affect NFCC or scores for either the conspiracy or attribution measure. Whist these variations are clearly important in considering the broader phenomenon of beliefs in conspiracy theories, the scope of the present study required a clear focus on specific aspects of the psychological processes underpinning such beliefs. Hence the empirical focus was on a particular age group and broadly homogeneous white, middle class student sample.

### Method

#### Design

A correlational design examined the relation between beliefs in real world conspiracy theories and the likelihood of attributing events in a fictitious (or novel) scenario to a conspiracy. Other measures included in the analysis were: feelings of alienation, authoritarian-rebellion attitudes, levels of close interpersonal trust and NFCC.

#### Participants

Thirty participants (15 males, 15 females, mean age 22 years) were undergraduates attending a university in London, United Kingdom. All participants were volunteers. All but one of the participants described their ethnicity as white British (the other was a British subject of Indian origin). No exclusion variables were employed.

#### Materials and procedure

Participants were given an information sheet that included a list of generic questions and 6 attitude scales. The first scale was an 8-item Beliefs in Conspiracy Theories scale (BICT, Appendix 1). The second scale was a 20-item Alienation scale (Ray, 1982). The third scale was an adapted version of Kohn's (1972) Authoritarian- Rebellion scale (however, items 9, 12, 16, and 20 were altered to exclude questions relating exclusively to Canadian participants). The fourth scale was Rempel et al.'s, 1985 17-item Close Interpersonal Trust scale. The fifth scale was a 46-item NFCC scale (Webster and Kruglanski, 1994). The final measure was the attribution of the likelihood of a conspiracy theory in response to a fictitious vignette (see below).

The rating scales for all attitude measures (excluding the final attribution measure) were adjusted to a uniform 5-point Likert scale that ranged from 1 (strongly disagree) to 5 (strongly agree). On the attribution of likelihood scale a rating of 1 indicated a belief that a conspiracy theory was highly likely, whereas 5 indicated it was highly unlikely. The attribution of conspiracy measure asked participants to read a fictitious vignette reporting the death of a President of an unnamed nation in a plane crash (see Appendix 2). After reading the vignette, participants were asked to place a cross on a 5 cm line (ranging from left to right, 0 cm = very likely to 5 cm = very unlikely) responding to the statement; “How likely is it that there was a conspiracy behind the plane crash?”

Participants were given a booklet of the scales to complete in a pencil and paper test in a room on their own on campus. The questionnaire took around 20 min to complete.

Tests used only pre-existing measures that had good reliability. However, in order to ascertain the robustness of these measures for the present sample reliability tests were carried out on the present data. Initially all scales were shown to be reliable (alpha > 0.70), excluding the Authoritarianism-Rebellion scale. Following the removal of low scoring items from all scales the Authoritarianism-Rebellion scale achieved an acceptable reliability (alpha = 0.64), and the reliability of the other scales also improved. The final scale as a whole was also shown to be reliable (*N* = 30, items = 99, alpha = 0.86). Further reliability analysis was conducted on the NFCC scale by calculating the internal lie scale score. All items were found to be within the margin for inclusion (see again Webster and Kruglanski, 1994).

### Results

Pearson's correlations were conducted on the 30 participants' scores on the six attitude measures and results are shown in Table 1. We also conducted a correlation analysis between general BICT and the attribution of a conspiracy to explain a novel event. This correlation was not significant (Pearson, *N* = 30, *r* = −0.001, *p* = 0.997).

**Table 1:** **The correlation between scores on the alienation, authoritarian-rebellion, close interpersonal trust and NFCC scales, in relation to scores on the BICT and the attribution of conspiracy theory to explain a fictitious event**.

	**BICT (Real world conspiracy theories)**	**Attribution of conspiracy to explain a novel event**
Alienation	0.65^**^	−0.40^*^
Close interpersonal trust	−0.38^*^	0.04
Authoritarian-rebellion	0.28	0.10
NFCC	−0.05	−0.05

* p < 0.05;

** p < 0.001.

### Discussion

Contrary to expectations and some previous research (e.g., Swami, 2012) we found no relation between authoritarianism and either beliefs in conspiracy or the tendency to invoke a conspiracy theory to explain an unfamiliar event. However, as predicted, there was a negative correlation between close interpersonal trust and beliefs in “real world” (i.e., not the hypothetical, novel event) conspiracy theories. As others have found before (e.g., Goertzel, 1994) individuals with low levels of interpersonal trust tend to have higher levels of belief in conspiracy theories, probably because they are less inclined to believe common, standard or widely held accounts. However, there was no correlation between levels of interpersonal trust and the attribution of a conspiracy theory to explain the unfamiliar event. Thus, individuals with low levels of trust may be less trusting of “official” accounts relating to real events, but low trust does not predispose people to attribute a conspiracy theory to a new event. This is an important finding because it suggests that whether or not we attribute a conspiracy theory to explain an event is initially unaffected by levels of interpersonal trust, but that over time trust may be a factor in whether or not beliefs in a conspiracy endure. In this respect the initial attribution of a conspiracy to explain an event may be a consequence of simple heuristic processes associating certain events with certain types of cause (see Leman and Cinnirella, 2007). However, as evidence is presented those with low levels of interpersonal trust may be more inclined to maintain beliefs in a conspiracy, whereas others allow those beliefs to diminish in light of subsequent evidence.

Both BICT and the attribution of conspiracy to a novel event correlated significantly with alienation scores. Alienation was also identified as a correlate of BICT. Alienation and the associated constructs of powerlessness and anomie have consistently been associated with BICT (Crocker et al., 1999). This suggests that BICT may stem, at least in part, from feelings of dislocation from society and social institutions. Alienation and anomie may also account for the interesting finding that BICT are higher in ethnic minority individuals (Crocker et al., 1999; Parsons et al., 1999; Bird and Bogart, 2003) because these are groups who, traditionally, have not been involved in government and other business, political and social institutions, and hence feel disconnected from authoritative decision-making processes.

Contrary to previous research (e.g., Swami et al., 2011) that has found an association between beliefs in different conspiracies, our present study found no correlation between beliefs in real world conspiracies and the likelihood of attributing a conspiracy to explain a novel event. This may be a consequence of the exploratory nature of the present study and low sample size. It may be a consequence of national differences (UK vs. the Austrian sample used in Swami et al.'s study 2 (2011), which included a fictitious example of a conspiracy theory involving the Austrian “inventor” of the drink Red Bull). Additionally, attributing a conspiracy to explain a fictitious event (constructing a conspiracy account) is a rather different matter, psychologically, from believing in a conspiracy account that others have already presented or that relates to an existing or actual event. Different types of event or theory may inspire or provoke different sorts of belief. Thus, an alternative explanation is that the decontextualized hypothetical (fictitious) scenario presented to participants here is a different type of stimulus compared with real world conspiracy beliefs. Thus, many conspiracy theories may stem from the same sense of disengagement with social institutions and authorities, or correspond to a particular set of political beliefs. And the correspondence between real world beliefs may be a consequence of their sharing a common “stem,” whereas our hypothetical scenario did not readily lend itself to any particular background story, context, or set of existing socio-political beliefs. Future research can help to establish what common features of conspiracy theories underpin such attributions.

The present findings point to the importance of individual and social factors in mediating levels of belief in conspiracy theory. However, the main motivation for the first study was to establish if there was any relationship between NFCC and BICT. As predicted, there was no such relationship. In other words, high levels of belief in conspiracy theory are not associated with participants' NFCC. However, although we predicted no relation between NFCC and conspiracy beliefs, other research has suggested that related or overlapping concepts may and may not be associated with such beliefs. For instance, Abalakina-Paap et al. (1999) found no association between individuals' tolerance of ambiguity and beliefs in conspiracies. On the other hand, Swami and Coles (2010); Swami et al. (2011); Swami (2012) found a positive relation between the big five trait of openness and BICT. Openness would appear to be negatively related to NFCC. However, it may be possible that openness characterizes an open-minded approach to unconventional views rather than to all views. As such, those who are less likely to accept official accounts (the status quo) may tend toward conspiracy theories. Thus, NFCC picks out a different feature of cognitive style that is independent of a societal consensus or socio-conventional thinking.

## Study 2

The first study found no relationship between NFCC and BICT, or the attribution of likelihood of a conspiracy theory to explain a novel or fictitious scenario. However, findings from the first study indicate that trust may be a factor in terms of whether conspiracy beliefs endure or diminish over time, perhaps as people come to scrutinize evidence. NFCC also influences the ways in which evidence is evaluated or scrutinized. Specifically, several studies have found that a high NFCC leads to less scrutiny of evidence and a desire to reach a decision quickly, whereas a low NFCC leads to more scrutiny (Ford and Kruglanski, 1995; De Dreu et al., 1999; Klein and Webster, 2002). In our second study we sought to establish how, if at all, NFCC relates to the ways in which evidence is evaluated in respect of BICT.

In the second study, a new group of participants was asked to read the same vignette describing the death of a President in a plane crash that was used in study 1 (see again Appendix 2). Again, participants were asked to attribute the likelihood that the death was the result of a conspiracy. However, after this participants were asked to read additional evidence that either supported a conspiracy explanation for events, or did not support this account. In addition to different forms of evidence, NFCC was also experimentally manipulated to be lower for some participants. After reading this evidence, and under different NFCC conditions, participants again completed the attribution measure. Study one suggested that BICT may diminish over time or in light of scrutiny of subsequent evidence. Therefore, in this second study, participants completed the attribution measure once again, 2 h later. Participants' levels of belief in real world conspiracy theories were again measured using the BICT.

Following Ford and Kruglanski (1995) NFCC was manipulated by varying the level of accountability to which participants were subjected. This manipulation produced two groups of participants. In the first, no specific additional instructions were given. However, in the second (the high accountability group), participants were informed that they might be required to give an explanation of their decision to a large group of eighty peers. In this second group NFCC is lowered because the need for greater accountability leads individuals to scrutinize their judgments and beliefs more closely. In this case participants would be inclined to think more carefully about (or scrutinize more systematically) their decision to attribute the likelihood of a conspiracy theory to explain a fictitious event. Whilst the association between accountability and NFCC was not tested in this study, previous research has shown that accountability manipulations such as this consistently lower NFCC (see again Ford and Kruglanski, 1995).

In light of findings from study one, it was predicted that there would be no difference between groups when making the initial attribution before manipulating differences in NFCC. However, a main effect of evidence type (pro- or anti-conspiracy) was expected: it was predicted that those reading evidence *supporting* a conspiracy theory would rate a conspiracy theory explanation as more likely after reading the evidence, whereas those reading evidence *against* a conspiracy theory would rate the conspiracy as less likely after reading this evidence.

An interaction was predicted between evidence condition (pro- and anti-conspiracy) and NFCC (normal and low) groups when attributing the likelihood of a conspiracy after differences in accountability had been introduced. Specifically, it was anticipated that NFCC would magnify the influence of evidence type: when NFCC was lowered (high accountability) those reading pro-conspiracy evidence would be even more likely to attribute a conspiracy than those for whom there was no change in NFCC, and similarly for those reading anti-conspiracy evidence. This prediction relates to the theory that lowered NFCC, produced in this instance from increased accountability, allows for systematic processing of information (Klein and Webster, 2002). This in turn promotes assimilation of evidence and encourages attitude change, and also relates to research showing that NFCC levels mediate the extent to which evidence is re-interpreted (De Dreu et al., 1999).

### Method

#### Design

A mixed experimental design was employed. There were three independent variables. The first, a between groups (pseudo-independent) variable, was BICT and was measured using the BICT (see Study 1). For the purposes of analysis participants were divided into two groups around the midpoint of the scale (20 out of 40 maximum score) with high and low levels of belief. This division into high and low scores distinguished participants based upon features of the scale itself and constituted a sensible approach to distinguishing groups around the scale's midpoint. The second independent variable was level of NFCC. This was either normal or low and was again a between groups variable determined through random allocation of participants to either high or low accountability conditions. A third independent variable was a between groups variable and was the evidence condition: either pro-conspiracy or anti-conspiracy theory evidence.

The dependent variables were repeated measures of the attribution of the likelihood of a conspiracy theory at three different time points: first before reading evidence, second after reading the evidence, and third 2 h after reading the evidence.

#### Participants

Eighty-six participants were involved in the study. Participants were students at a university in South East England, United Kingdom and were recruited on a voluntary basis during a class that they were all attending. There were 79 women and 7 men, average age 21 years. In terms of ethnicity, 15 described their ethnicity as South Asian (Indian, Bangladeshi, Pakistani), 1 as Black (African-Caribbean) and 70 as white (European).

#### Materials and procedure

Participants were given a questionnaire pack that included an information sheet, a consent form, the vignettes, several attribution scales and finally the BICT. Altogether there were four different versions of the questionnaire distributed at random to participants in the class. In all versions, after the introductory questions and consent, participants read the vignette and rated the likelihood of a conspiracy theory to explain events. The following sections differed depending on the accountability (NFCC; high or low) and evidence (pro- or anti-conspiracy) condition. In one, participants were given instructions inducing high accountability and then read pro-conspiracy evidence and, in another, high accountability instructions and anti-evidence. In another, they were given no instructions about accountability and pro-conspiracy evidence and in another, no instructions about accountability and anti-conspiracy evidence. Evidence statements (pro- and anti-conspiracy) are given in Appendix 3.

Accountability was manipulated by including in the instructions written in the questionnaire booklet that five individuals would be required to stand up in front of the rest of the class (of 80 peers) and justify their response. For the no accountability condition, there were no such instructions. In the event, participants were not required publicly to justify their responses. Participants were fully debriefed at the end of the session.

The attribution of conspiracy question was the same and asked three times. First, before reading evidence and participants read any accountability instructions, second, immediately after reading the evidence and third, after 2 h. A 2-h delay was chosen for both conceptual and practical reasons. From a practical perspective, this was the longest period participants could reasonably be asked to remain without discussing their ratings with other participants. From a conceptual perspective, 2 h is widely considered to be adequate time to observe changes in judgment, attitudes, and reasoning, whilst constituting a meaningful separation time between testing sessions.

Each time, as in study 1, participants were asked to place a cross on a 5 cm scale indicating how likely they felt it was that “…a conspiracy caused the plane crash.” A score was calculated by measuring the distance in millimeters along the line. The higher score (50) indicated that participants thought a conspiracy explanation unlikely. A lower score, that they found a conspiracy attribution very likely. During the 2 intervening hours between the penultimate and final time the question was asked, participants were involved in a class and were not able to discuss the tasks with one another.

#### Reliability of measures

Reliability analysis conducted on the BICT scale indicated good reliability (*N* = 86, alpha = 0.61).

### Results

An initial related *t*-test found no difference between individuals with high and low levels of belief and the attribution of a conspiracy to explain a novel event at time 1 (*t*1 only), *t*_(85)_ = 0.65, *p* = 0.27. Subsequently, a 2 × 2 × 2 (BICT × evidence condition × NFCC condition) repeated measures ANOVA was conducted on the attribution score at time *t*1, *t*2, and *t*3.

In terms of within-subjects measures, there was a main effect of BICT [*F*_(1, 76)_ = 8.62, *p* < 0.01, partial η^2^ = 0.10]. *Post-hoc* simple effects tests found a significant difference only at *t*3, *t*_(82)_ = 2.52, *p* < 0.05, where those with high levels of belief in real world conspiracy theories rated a conspiracy explanation more likely for the fictitious event than those with low levels of belief in real world conspiracy theories. Related *t*-tests revealed significant differences between *t*1 and both *t*2 and *t*3 for those with low levels of belief in conspiracies: *t*1 vs. *t*2, *t*_(32)_ = 2.19, *p* < 0.05; *t*1 vs. *t*3, *t*_(32)_ = 2.75, *p* < 0.01). There were no significant differences for a similar comparison between attribution scores at different times for those with high BICT. Table 2 shows mean ratings for the likelihood that a conspiracy theory explains the event best by BICT.

**Table 2:** **Mean attribution ratings (standard deviations in parentheses) for the likelihood that a conspiracy theory explains the event best by beliefs in conspiracy theories**.

	**Attribution of likelihood of a conspiracy to explain fictitious event (0 = very likely, 50 = very unlikely)**		
**Beliefs in conspiracy theories**	***T*1**	***T*2**	***T*3**
High (*N* = 53)	22.23 (8.72)	21.32 (13.17)	20.25 (9.88)
Low (*N* = 33)	20.12 (8.81)	25.30 (13.06)	25.91 (9.88)

There was also a strong effect of evidence condition on the repeated measure, *F*_(1, 76)_ = 19.87, *p* < 0.001, partial η^2^ = 0.21. There was a significant difference between evidence conditions at both *t*2, *t*_(84)_ = 9.30, *p* < 0.001, and *t*3, *t*_(82)_ = 4.63, *p* < 0.001. Related *t*-tests found significance for comparisons across all times for those reading pro-conspiracy evidence: *t*1 vs. *t*2, *t*_(41)_ = 8.04, *p* < 0.001; *t*2 vs. *t*3, *t*_(39)_ = 3.05, *p* < 0.001; *t*1 vs. *t*3, *t*_(39)_ = 4.31, *p* < 0.001. Similarly, all comparisons for those reading anti-conspiracy evidence were significant: *t*1 vs. *t*2, *t*_(43)_ = 7.91, *p* < 0.001; *t*2 vs. *t*3, *t*_(43)_ = 3.48, *p* < 0.001; *t*1 vs. *t*3, *t*_(43)_ = −3.52, *p* < 0.001. Table 3 shows mean ratings for the likelihood that a conspiracy theory explains the event best by evidence condition.

**Table 3:** **Mean attribution ratings for the likelihood that a conspiracy theory explains the event best by beliefs in conspiracy theories and evidence condition**.

	**Attribution of likelihood of a conspiracy to explain fictitious event (0 = very likely, 50 = very unlikely)**		
**Evidence condition**	***T*1**	***T*2**	***T*3**
Pro-conspiracy (*N* = 42)	21.86 (7.84)	13.29 (7.95)	17.55 (6.97)
Anti-conspiracy (*N* = 44)	21.00 (9.65)	31.98 (10.45)	26.95 (10.99)

There were two effects between subjects. Firstly, as might be expected from inspecting the means in Table 3, there was a main effect of evidence type, *F*_(1, 76)_ = 35.24, *p* < 0.001, partial η^2^ = 0.32. As was anticipated, those reading pro-conspiracy evidence were more inclined, across the task, to consider a conspiracy likely than those reading anti-conspiracy evidence across the task.

**Figure 1:**
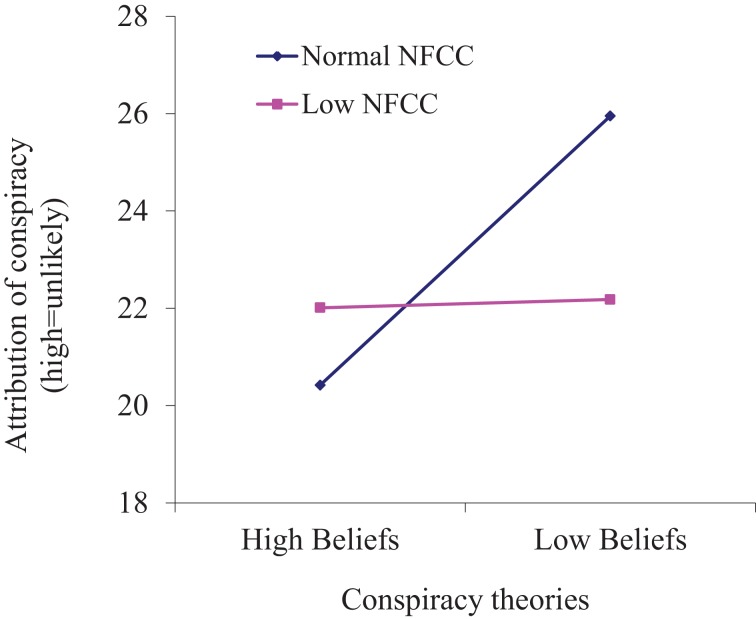
**Mean likelihood attribution score at time *t*2 (after reading evidence) by NFCC and beliefs in conspiracy theories**.

Secondly, there was a weak but significant interaction between BICT, the attribution of conspiracy theories to a novel event (at *t*2), and NFCC condition, *F*_(1, 76)_ = 6.34, *p* < 0.05, partial η^2^ = 0.02. Figure 1 shows the interaction. Those with normal NFCC (that is, in the low accountability condition) tended to make attributions, after reading the evidence, that were more concordant with their levels of belief in real world conspiracy theories: those with high levels of belief rated a conspiracy explanation more likely than those with low levels of belief. However, individuals with high and low levels of BICT made broadly similar attributions of the likelihood of a conspiracy when NFCC was lowered (high accountability condition).

Finally, three separate 2 × 2 × 2 (evidence × NFCC × BICT) ANOVAs were conducted on the attribution scores at each separate time interval (*t*1, *t*2, and *t*3). These revealed significant effects of evidence condition at *t*2, *F*_(1, 76)_ = 94.52, *p* < 0.001, partial η^2^ = 0.55. and *t*3, *F*_(1, 76)_ = 23.76, *p* < 0.001, partial η^2^ = 0.24, but not at *t*1. In both cases, the evidence type affected the attribution score; those reading pro-conspiracy evidence rated a conspiracy as a more likely explanation, those reading anti-conspiracy evidence rated it as less likely. There was also a main effect of BICT at *t*3 only, *F*_(1, 76)_ = 5.17, *p* < 0.05, partial η^2^ = 0.06. There were also significant NFCC × BICT interactions at *t*2, *F*_(1, 76)_ = 7.20, *p* < 0.01, partial η^2^ = 0.09, and at *t*3, *F*_(1, 76)_ = 4.28, *p* < 0.05, partial η^2^ = 0.05, but not at *t*1. Both of these interactions mirrored that from the between-subjects interactions from the repeated measures MANOVA (see again Figure 1): lowered NFCC appeared to mollify the impact of prior BICT.

However, we found no relationship between NFCC and evidence condition in terms of the attribution measure, *F*_(1, 76)_ = 1.48, *p* = 0.76.

### Discussion

At baseline, before reading evidence or information relating to high or no accountability (NFCC) conditions, levels of NFCC and evidence condition did not relate to the initial attribution of a conspiracy theory to explain the fictitious event. However, again as predicted, after reading evidence there were significant and strong effects associated with evidence type: specifically, participants who read evidence that supported a conspiracy theory rated a conspiracy explanation as more likely. Those who had read evidence that undermined a conspiracy theory account rated a conspiracy explanation less likely. These effects are consistent with the observation that with ambiguity mere exposure leads to influence (Eagly and Chaiken, 1993), and in part echo Newheiser et al. (2011) experimental demonstration that exposure to counter-conspiracy evidence can result in lowered belief in the conspiracy.

A further prediction was that there would be an interaction between level of NFCC and evidence type and in particular that lower levels of NFCC would be associated with greater influence of evidence type. This relationship was predicted because lowering NFCC is known to induce more systematic processing of information and, given that the evidence presented to participants was either clearly supporting or clearly undermining a conspiracy theory, this more systematic processing should lead to more dramatic influence when rating the likelihood of a conspiracy theory to explain the fictitious event. However, this prediction was not supported. Indeed, a significant interaction suggests a more complex pattern of relations involving NFCC, evidence and levels of belief in real world conspiracy theories.

Our analysis also indicated a difference between those with high and low levels of belief in conspiracy theories in terms of their ratings of likelihood after reading evidence of either sort and after a 2 h delay. Additional *post-hoc* tests showed this effect to be attributable to changes in the low beliefs group after reading evidence.

One explanation is that individuals with low levels of belief in conspiracy theories were more responsive to anti-conspiracy evidence and thus evaluated this evidence more favorably than pro-conspiracy evidence. However, it remains unclear why those with high levels of belief in conspiracy theories did not show a similar bias (however, although not significant, there was a trend in this direction). A further explanation fits with other findings (Leman, 2007; Leman and Cinnirella, 2007) which found that individuals with low levels of belief may be more trusting of the veracity of reported facts than those with high levels of belief in conspiracy theories (in the absence of further evidence the inference or attribution of conspiracy itself remained unaffected by levels of belief in conspiracy theories). If this is the case, those with low levels of belief in conspiracy theories may simply be more easily influenced by evidence *per se* and this, combined with biases in evaluating evidence, leads to the significant effects in the low beliefs group seen in the present study.

Effects of evidence condition were very strong, and were certainly much stronger than any effects of NFCC or BICT. However, on the face of it these evidence effects were relatively short-lived, and although they were still present, tended to diminish after a 2-h interval when ratings were taken again on the likelihood (attribution) measure. This contrasts, as we have seen, with what appears to be a less immediate but more enduring influence of BICT on ratings.

Finally, an interaction between NFCC and BICT points to a complex set of relationships between the variables in terms of the attribution of likelihood of a conspiracy to explain a fictitious event. When NFCC was lowered there was very little difference between likelihood ratings from participants with high and low levels of belief in conspiracy theories. However, for participants not in the high accountability condition (normal NFCC) individuals with high BICT tended to rate a conspiracy more likely, whereas those with low beliefs tended to rate a conspiracy less likely after reading the evidence. Once again, this interaction holds true only after reading evidence but is not affected by the *type* of evidence read. And again, this suggests that individuals' BICT may incline them to process or evaluate evidence in a manner that is consistent with their existing BICT.

Importantly though, the effects of BICT are nullified by lowering NFCC. With lower NFCC individuals are more motivated to both attend to and scrutinize in more detail the evidence (Klein and Webster, 2002). Hence we see rather more cautious ratings of likelihood in the low NFCC group, reflecting that both pro- and anti-conspiracy evidence is examined in more detail than in the normal NFCC group. This finding is consistent with research in the schema literature, which indicates that the goal of accuracy (which may well have been activated in the low NFCC manipulation) makes people remember and process more carefully schema-relevant information, and even schema-inconsistent information (see Fiske and Taylor, 1991 for an overview).

## General discussion

Our two studies examined the relationship between BICT, NFCC, and the ways in which evidence is evaluated in respect of a fictitious event that may (or may not) have been attributable to a conspiracy. Consistent with previous work (e.g., Abalakina-Paap et al., 1999; Leman, 2007; Swami, 2012), our first study found that an individual's sense of alienation correlated with their levels of belief in conspiracy theories. Also consistent with previous evidence (Goertzel, 1994) was a correlation between low levels of interpersonal trust and BICT.

Taken together the present findings extend our understanding of social, personality and cognitive factors associated with BICT. In this regard study 2 identified a complex relationship between existing levels of belief in conspiracy theories, NFCC and the evaluation of evidence. Specifically, existing BICT do not appear immediately to affect an individual's attribution of a conspiracy theory to explain a novel (fictitious) event. But, over time individuals display a tendency to assimilate new events in a manner that is consistent with existing beliefs. This connects with (Wood et al., 2012) research showing that individuals have broad general beliefs—monological belief systems—in conspiracy that can make them endorse new conspiracy theories. It also connects with clinical research (e.g., Mackay et al., 2006, 2007) indicating that certain delusional beliefs may be connected with NFCC in sometimes complex ways.

However, the present results appear to sit uneasily with other research (e.g., Swami et al., 2011) which found that beliefs in real world conspiracy theories were correlated with the likelihood of attributing a theory to a ficitious, novel event. As we suggested, it may be that specific types of event inspire different types of reaction and more research is certainly needed to articulate the relationship between events and conspiracy theories. The reason for the mismatch between research findings here may, therefore, connect back to earlier research (Leman and Cinnirella, 2007) which identified features of an event as a significant component in creating conspiracy theories. Big, sudden, or tragic events may, initially, lead more people to adopt a conspiracy explanation whereas conspiracies to do with public health and the motivations of businessmen may tap into existing beliefs about the world more quickly for some than others.

Lowering NFCC (increasing accountability and hence giving participants a greater motivation to scrutinize the evidence and justify their rating) appeared to cancel out the influence of existing BICT. This finding is consistent with research on the effects of accuracy motivations in schematic processing and on stereotypic processing, with all of these research areas demonstrating that when accuracy becomes important to the actor, it can overcome tendencies toward processing information in a heuristic manner and encourage more systematic processing. This latter finding also suggests that those who took a less systematic (more heuristic) approach to evaluating any evidence were more likely to end up with an account that was more consistent with their previous beliefs.

While biases in the evaluation and assimilation of evidence may be part of the story, the relationship between BICT and evidence may be more complex still in real-world situations for at least two reasons. Firstly, it may not be merely processing of information but also the search for information (or evidence) that is subject to biases (Lord et al., 1979; Klayman and Ha, 1987; McHoskey, 1995). In this respect, a hard-nosed conspiracy theorist may seek out (or regard as legitimate) only the evidence that conforms to a particular view. In a similar vein, a hard-nosed anti-conspiracy theorist may not only reject evidence that points toward a conspiracy theory account but also spend more time and devote more psychological resources to seeking out evidence that undermines a conspiracy account.

Secondly, the present study explored attributions relating to the likelihood of a conspiracy theory to explain a novel, fictitious event. Whilst such an approach makes experimental study possible and reduces the possibility of un-measured variables creating noise in the data, it removes context from the decision-making process. This final point is most clearly illustrated by findings from the first study that identified alienation and low levels of interpersonal trust as correlating with BICT. Whilst the negative correlation between interpersonal trust and BICT points toward a role for personality factors (see again Goertzel, 1994), the consistent finding across these and other studies of a strong relationship between feelings of alienation and BICT suggests, again, that broader social processes are also at play (e.g., Crocker et al., 1999). Indeed, the link between conspiracy theories and feelings of alienation suggests intriguing parallels with inter-group phenomena and aspects of individuals' social identities. For example, defensive attributions and complex intergroup processes may lie behind the adoption by some Muslims of 9/11 conspiracy theories. In this respect adoption and endorsement of conspiracy theories could ultimately become a mechanism for expressing social identity under circumstances where adoption of particular conspiracies is deemed to be normative for a group. Thus, potentially fertile ground for future research would be to investigate the degree to which levels of interpersonal trust and aspects of an individual's social identity may predispose individuals to high levels of belief in conspiracy theories. Any such research would benefit from using real-world conspiracy theories that resonate with the social identities of participants. In addition, there may be societal level forces which are acting to make conspiracy theories more popular amongst certain populations, and these need to be considered an important backdrop to the socio-psychological processes involved in conspiracy beliefs (Aupers, 2012).

### Conflict of interest statement

The authors declare that the research was conducted in the absence of any commercial or financial relationships that could be construed as a potential conflict of interest.

## Appendix 1

### Beliefs in conspiracy theories (BICT)

There was no conspiracy involved in the assassination of John. F. Kennedy. (-)

The European Union is trying to take control of the United Kingdom.

Princess Diana's death was an accident. (-)

Governments are suppressing evidence of the existence of aliens.

The AIDS virus was created in a laboratory.

The attack on the Twin Towers was not a terrorist action but a governmental conspiracy.

The American moon landings were faked.

A government exercise was behind the suicide at Jones Town.

NOTES:

1. Participants are asked to rate their beliefs in a 5-point scale (1, strongly disagree; 2, disagree; 3, don't know; 4, agree; 5, strongly agree)
2. (-) items are reverse scored
3. Total score (including reverse items) indicates levels of belief in conspiracy theories

## Appendix 2

### Attribution of a conspiracy theory to a novel (fictitious) event

Vignette

### News report: plane crash kills leading political figure

An investigation is under way after a 20-seater plane, carrying Sir——, crashed killing all of five people on board. The accident happened at around 1pm yesterday although emergency services arrived at the scene of the crash only after at 4.25 pm. A police spokesman said: “A full air accident investigation has been launched and at this time we believe that the crash was caused by mechanical failure.” He also stated that the plane was believed to have taken off from the —- area, but refused to reveal the intended destination of the plane. A senior duty officer with the —– Ambulance Service said: “I was down there this afternoon and it looked pretty bad. One eyewitness reported: everything seemed fine but then there was a bang and it nose-dived.”

It is understood that, prior to the incident, Sir —– was under 24 h protection in light of the upcoming election and that all possible safe guards had been put in place to ensure his safety. A source has also revealed that the journey was initially planned to be by rail and plans were switched at the last minute due to safety concerns. Political figures close to the former head of the opposition described the incident as shocking and devastating for the party. Also killed were three security agents and Sir —–'s press secretary.

## Appendix 3

### Evidence for (pro-conspiracy) and against (anti-conspiracy) conspiracy theory account for the fictitious event

Pro-conspiracy:

The accident investigation report could not identify a cause for the crash.

Some interested parties have expressed concern that the crash may not have been an accident.

The plane had been given a full engineering check only the day before the crash, and was judged to be in excellent working order.

The emergency services arrived late because they had received contradictory evidence from anonymous witnesses concerning the location of the plane.

The “safety concerns,” which had necessitated the change in travel plans, related to intelligence suggesting that an assassination attempt on Sir—– was imminent.

Anti-conspiracy:

The accident investigation report cited mechanical failure as the cause of the crash.

All interested parties were satisfied that the crash had been an accident.

Three months prior to the crash an identical fault had been detected on another plane of the same model, type and make.

The late arrival of the emergency services had been due to the restricted information concerning the location of the plane.

The security threat, which had necessitated the change in travel plans, was deemed to be unfounded.
